# Clinical Management of New-Onset Atrial Fibrillation in COVID-19 Patients Referred to a Tertiary Cardiac Arrhythmia Center after Hospital Discharge

**DOI:** 10.3390/jcm11195661

**Published:** 2022-09-26

**Authors:** Marco Schiavone, Fabiola B. Sozzi, Alessio Gasperetti, Cecilia Gobbi, Elisa Gherbesi, Lucia Barbieri, Roberto Arosio, Gianfranco Mitacchione, Filippo Toriello, Andrea Faggiano, Maurizio Viecca, Giovanni B. Forleo, Stefano Carugo

**Affiliations:** 1 Cardiology Unit, Luigi Sacco University Hospital, Via Giovanni Battista Grassi 74, 20157 Milan, Italy; 2 Department of Systems Medicine, University of Rome Tor Vergata, 00133 Rome, Italy; 3 Cardiology Unit, Fondazione IRCCS Ca’ Granda Ospedale Maggiore Policlinico, 20122 Milan, Italy; 4 Division of Cardiology, Johns Hopkins University, Baltimore, MD 21218, USA; 5 Interventional Cardiology, Saint Martin Private Hospital Center, 14000 Caen, France

**Keywords:** atrial fibrillation, COVID-19, cardiac arrhythmias, rhythm monitoring, catheter ablation

## Abstract

Background: Available reports on the post-discharge management of atrial fibrillation (AF) in COVID-19 patients are scarce. The aim of this case series was to describe the clinical outcomes of new-onset AF in COVID-19 patients referred to a tertiary cardiac arrhythmia center after hospital discharge. Methods: All consecutive patients referred to our center for an ambulatory evaluation from 18 May 2020 to 15 March 2022 were retrospectively screened. Patients were included in the current analysis if new-onset AF was diagnosed during hospitalization for COVID-19 and then referred to our clinic. Results: Among 946 patients, 23 (2.4%) were evaluated for new-onset AF during COVID-19. The mean age of the study cohort was 71.5 ± 8.1 years; 87.0% were male. Median time from COVID-19 discharge and the first ambulatory evaluation was 53 (41.5–127) days; median follow-up time was 175 (83–336) days. At the in-office evaluation, 14 (60.9%) patients were in sinus rhythm, and nine patients were in AF. In 13.0% of cases, oral anticoagulation was stopped according to CHADS-VASc. Eight patients in AF were scheduled for electrical cardioversion; one patient was rate-controlled. Four patients were treated with catheter ablation (CA) during follow-up. Two post-cardioversion AF recurrences were detected during follow-up, while no recurrences were diagnosed among patients who underwent CA. Conclusion: Our data suggest that AF may not be considered as a simple bystander of the in-hospital COVID-19 course. Management of new-onset AF in post-COVID-19 patients referred to our clinic did not significantly differ from our usual practice, both in terms of long-term oral anticoagulation and in terms of rhythm control strategy.

## 1. Introduction

In addition to an established respiratory involvement [1,2], a significant myocardial injury in Coronavirus disease (COVID-19) has been ascertained, often triggered by macro- and microthrombosis, as well as by direct cardiac damage [3,4,5,6,7,8,9,10]. Indeed, as a consequence, cardiac arrhythmias and acute coronary syndromes (ACS) have been widely reported as potential issues, especially in hospitalized patients, often worsening COVID-19 patients’ prognosis [11,12,13,14,15,16,17,18]. In particular, in up to 44% of COVID-19 patients admitted to the intensive care unit (ICU), brady- and tachyarrhythmias were detected, and thus, up to 17.6% of admitted patients experienced atrial fibrillation (AF) [4,19]. Of the total 9564 patients included in this study from Mountantonakis et al., 1687 (17.6%; 95% CI 16.9–18.4%) experienced AF during hospitalization; among these, 1109 patients (65.7%; 95% CI 63.4–68.0%) had new-onset AF [19]. Moreover, it has been progressively highlighted that AF, and particularly new-onset AF, is an independent predictor of in-hospital mortality, when compared to patients with a history of AF [19]. Interestingly, it has emerged that the presence of structural heart disease was not associated with a higher risk of in-hospital mortality in patients developing AF, suggesting that new-onset AF is independent from any previous cardiac disorder, either structural or arrhythmogenic [19]. Nevertheless, if it has been reported that many people with mild–moderate disease recover within 2 weeks, a certain amount of patients do not return to baseline even after 14–21 days, and possibly develop the so-called “post-COVID-19 syndrome”, with a non-negligible involvement of the cardiovascular system [20]. Several longitudinal studies on the Post-Acute COVID-19 Syndrome have been summarized in a review from Dixit et al. [21], who have reported that the rate of COVID-19 cardiovascular patients’ rehospitalizations may significantly vary, being up to 15.1% [22]. Indeed, the rise in post-COVID 19 cardiac manifestations is expected to have detrimental consequences for the prevalence and economic projections of the cardiac patient, with atrial tachyarrhythmias and AF representing a significant part of the entire spectrum. To date, available reports on new-onset AF in COVID-19 are mainly focused on the arrhythmic in-hospital management; therefore, the aim of this case series was to describe the clinical outcomes of new-onset atrial fibrillation in COVID-19 patients referred to a tertiary cardiac arrhythmia center after hospital discharge.

## 2. Methods

### 2.1. Study Population

All consecutive patients referred to our tertiary cardiac arrhythmia center (Luigi Sacco University Hospital, Milan, Italy) for an ambulatory evaluation from 18 May 2020 (date of the first lockdown ending in Italy) to 15 March 2022 were retrospectively screened. Patients were included in the current analysis if new-onset AF was diagnosed during hospitalization for COVID-19 and then referred for a subsequent clinical evaluation in our arrhythmia center clinic after discharge. Patients with a previous history of AF were excluded from the current analysis. Patients’ follow-up and further clinical evaluations or intervention were left to physicians’ and patients’ choice.

### 2.2. Data Collection and Study Outcomes

Baseline demographic and clinical characteristics, as well as patient treatment and clinical outcome data, were obtained from in-hospital electronic medical records. All data were retrospectively analyzed. First-diagnosed or new-onset AF was defined according 2020 European Guidelines [23]. A confirmed case of COVID-19 was defined by a positive result on a reverse-transcriptase polymerase chain reaction (RT-PCR) assay performed on a nasopharyngeal swab, according to the World Health Organization (WHO) guidelines. This study was conducted in accordance with the Declaration of Helsinki. Outcomes of the current analysis were:

- Clinical management of new-onset AF in the study cohort;
- AF recurrence after physician intervention in the follow-up period.

### 2.3. Data Collection and Study Outcomes

Categorical variables were reported as counts (percentage). Normality of distribution was tested for all continuous variables using a Shapiro–Wilk test. Continuous variables are reported as mean standard deviation (s.d.) or as median (IQR, interquartile range) if normally or nonnormally distributed, respectively. All statistical analyses were performed using STATA version 14.0 (Stata Corp, College Station, TX, USA).

## 3. Results

### 3.1. Baseline Characteristics

Among 946 patients referred to our tertiary cardiac arrhythmia center for an ambulatory visit from 18 May 2020 to 15 March 2022, 23 patients (2.4%) were evaluated after hospital discharge for new-onset AF detected during COVID-19 hospitalization. Characteristics of the study cohort are reported in Table 1. Overall, the mean age of the entire study cohort was 71.5 ± 8.1 years; 20 patients (87.0%) were male. As for cardiovascular risk factors, 14 (60.9%) and 2 (8.7%) patients suffered from hypertension and diabetes, respectively, while, as for history of previous cardiac disease, two patients (8.7%) had a previous history of cardiomyopathy, with an ischemic etiology in both cases, resulting in heart failure (HF) with reduced ejection fraction in one case. When considering significant comorbidities, three patients (13.0%) showed vascular diseases, with a history of stroke/TIA in one case; two patients (8.7%) suffered from CKD. The median CHA2DS2-VASc score was 2 (1–3), while the median HAS-BLED score was 2 (1–2), with one patient having a previous history of significant bleeding (gastrointestinal).

### 3.2. Patient Management and Clinical Outcomes

All patients were admitted due to COVID-19 in our institution, with no patient needing intensive care unit (ICU) admission. During admission, in 16 (69.6%) cases, the arrhythmia terminated during admission for a spontaneous, pharmacological, or electrical cardioversion. Twenty-one (91.3%) patients were discharged on oral anticoagulation treatment (19 patients on direct anticoagulants (DOAC) and 2 patients on low-molecular-weight heparin), while in two cases, no long-term anticoagulation treatment was started. In 11 (47.8%) cases, an antiarrhythmic drug treatment was set up, mostly with amiodarone. Fifteen (65.2%) patients were implanted with an implantable loop recorder (ILR) to evaluate the arrhythmic burden; among those, 10 patients underwent ILR implantation during the in-hospital admission, while five patients were scheduled to implant an ILR after the post-discharge in-office evaluation.

**Figure 1:**
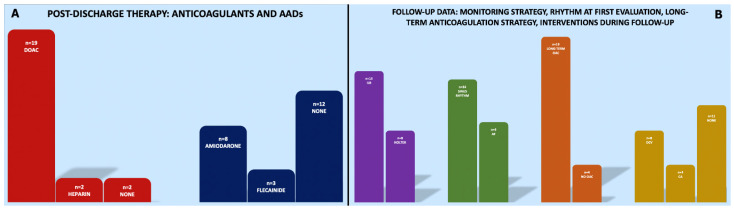
Clinical management of the study cohort: post-discharge therapy (**A**) and follow-up data (**B**). (**A**) Red bars summarize anticoagulant therapy at discharge: 19 patients were discharged on direct oral anticoagulants (OAC), 2 patients were discharged on heparin, and 2 patients were discharged without anticoagulation. Blue bars summarize antiarrhythmic drug (AAD) therapy at discharge: 8 patients were discharged on amiodarone, 3 patients were discharged on flecainide, and 12 patients were discharged without AADs. (**B**) Purple bar summarizes the rhythm monitoring strategy that was chosen at discharge: 15 patients were followed up with an implantable loop recorder (ILR), either implanted during admission or implanted during follow-up, while 8 patients were followed up with periodic Holter-ECG evaluations. Green bars summarize patients’ cardiac rhythms at the first in-clinic evaluation: 14 patients were in sinus rhythm, while 4 patients were in AF. Orange bars summarize clinical choices regarding OAC: in 19 patients, a long-term OAC strategy was started, while in 4 cases, OAC was terminated. Yellow bars summarize any intervention during follow-up: 8 direct cardioversions (DCV) and 4 catheter ablations (CA) were performed.

**Figure 2:**
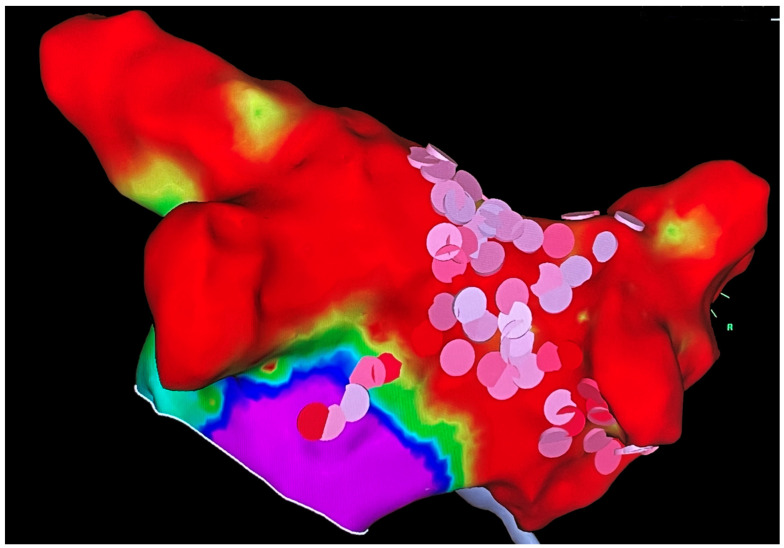
Three-dimensional bipolar voltage map of the left atrium after radiofrequency catheter ablation of persistent AF; pink dots indicate radiofrequency pulses on the posterior wall.

The median time from COVID-19 discharge to the first ambulatory evaluation was 53 (41.5–127) days. At the time of the in-office evaluation, 14 (60.9%) patients were in sinus rhythm, while nine patients were in AF. As per current guidelines for the management of AF [23], in three (13.0%) cases (all in sinus rhythm), oral anticoagulation was stopped (in all cases, at least 4 weeks after AF onset), with two patients showing CHADS-VASc score = 0 and one patient showing CHADS-VASc score = 1 (female sex). In 1 out of 2 patients who were not on anticoagulant therapy at discharge, a DOAC therapy was started in-office, due to CHADS-VASc score = 3; all patients on low-molecular-weight heparin were shifted to DOACs. Regarding rhythm control management, 8 out of 9 patients in AF were scheduled for an electrical cardioversion, while in one patient, a rate control strategy was chosen. Four patients were treated with catheter ablation (CA) during follow-up: one with a laser-balloon technique with pulmonary vein isolation (PVI) and three with radiofrequency CA (two patients underwent PVI, while one patient underwent PVI + posterior wall isolation due to persistent AF). During the follow-up time (median 175 (83–336) days), among the 14 patients who were in sinus rhythm at the first clinic evaluation, five patients experienced at least one AF recurrence. Moreover, two post-cardioversion AF recurrences were detected during follow-up, while no recurrences were detected among patients who were treated with CA. The clinical management of the study cohort is summarized in Figure 1. A three-dimensional bipolar voltage map of the left atrium after radiofrequency CA of persistent AF is shown in Figure 2.

## 4. Discussion

In this case series of patients referred to our tertiary cardiac arrhythmia for first-diagnosed AF in COVID-19, AF management was neither limited nor strictly related to the course of the disease itself. Indeed, 60.1% of the entire cohort were either in AF at the first post-discharge in-office evaluation or experienced at least one AF recurrence during follow-up. Moreover, 52.2% of the entire cohort was either cardioverted or underwent CA during follow-up, and in 87% of cases, an indication of long-term anticoagulation was given by the cardiac electrophysiologist. Therefore, new-onset AF had a significant impact on patients’ clinical status, and clinical management did not significantly differ from our usual practice, both in terms of long-term oral anticoagulation and in terms of the rhythm control strategy proposed to these patients.

### 4.1. Arrhythmogenesis in COVID-19 after the Acute Phase

Several reports have clarified how COVID-19 infection is related to an increased risk of cardiac arrhythmias by several pathophysiological mechanisms, such as myocardial injury (mainly due to hypoxia, ischemia, or direct viral damage) and extracardiac processes (cytokine storm or electrolyte imbalance), that may induce or precipitate cardiac arrhythmias, especially in patients with a pre-existing propensity [24,25]. The predominant COVID-19 manifestation is respiratory involvement, potentially increasing intracellular calcium and potassium levels, which, along with a hyperadrenergic tone, contribute to the development of early and late afterdepolarizations, as well as enhanced cellular excitability and electrical conduction velocity [26]. All these mechanisms that contribute to the arrhythmogenic mechanisms triggering AF are closely related to the acute phase of COVID-19, but it is not completely understood how they could be responsible for further recurrences or for AF maintenance. However, despite an insufficient understanding of the underlying anatomic and functional basis for AF, it is known that the vulnerable electrophysiological and/or anatomical substrate needed for AF maintenance did not completely match with the triggers needed to initiate AF. Notwithstanding, both parasympathetic and sympathetic stimulation play a role both in triggering and maintaining AF [27] and, in this regard, the emerging concept of COVID-19-induced dysautonomia, which has been linked with the post-COVID syndrome, significantly impairing cardiovascular homeostasis, as witnessed by a significantly lower heart rate variability (HRV) compared to healthy controls, may show a pivotal role also in AF maintenance and recurrences [28,29].

- Apart from hypoxia, SARS-CoV-2’s direct penetration into myocardial cells through the receptors of the angiotensin-converting enzyme-2 (ACE-2), as well as activation of virus-triggered CD8+ T lymphocytes, might result in myocardial injury, remodeling, and adverse cardiac outcomes, seen as subclinical or overt myocarditis [30]. Cellular damage, ionic imbalance, and gap junction dysfunction may result in early afterdepolarizations and delayed afterdepolarizations, along with reduced or increased conduction velocity and decreased refractoriness, increasing the likelihood of circus-type reentry [31]. These mechanisms are also of the utmost importance in triggering AF out of the acute phase since inflammation is known to be associated with recurrent AF through the involvement of cellular degeneration, apoptosis, and subsequent atrial fibrosis, which is extremely difficult to determine clinically [27].
- Myocardial ischemia, mostly due to a hyperinflammatory response, microvascular dysfunction, proatherogenic effects, and vasculitis, might induce significant myocardial sequelae, leading also to a non-transient endothelial dysfunction [32,33]. Indeed, a recent growing body of evidence links AF to atrial and systemic endothelial dysfunction. A postulated *liaison* between AF and endothelial dysfunction includes inflammatory or oxidative stress as well as common pathway biomarkers, which might feed a vicious cycle resulting in worse endothelial dysfunction and persistent AF [34].

Finally, when evaluating echocardiographic characteristics, only one patient showed significant (at least moderate) mitral regurgitation, while the LA volume index was overall within normal range. If it is true that this was a globally old cohort, the prevalence of a known cardiovascular disease was low, and this might be the best explanation for these findings. Nevertheless, also a subclinical “atrial cardiomyopathy” may have led these patients to be more susceptible to the development of new-onset AF than others. All these mechanisms, as well as individual susceptibility, might underpin AF onset and lead to recurrences, thus corroborating arrhythmogenesis also outside of the acute COVID-19 phase, as witnessed in our case series.

### 4.2. Long-Term Management of New-Onset AF Detected during COVID-19

As previously described, several risk factors have been regarded as triggers for the development of new-onset AF in COVID-19, not accounting for only the inflammatory state, as in septic patients. Indeed, several reports have determined how new-onset AF may occur in up to 20% of patients suffering from sepsis and 46% from septic shock, while it might aggravate the course of up to 30–50% of patients who underwent cardiothoracic surgery [35]. When considering indications for post-discharge oral anticoagulation in this setting, it has been described how most patients who developed new-onset AF were not anticoagulated at the time of discharge [35]. Indeed, although specific indications and guidelines have not been provided so far, most recent guidelines for AF highlight that in patients at risk of stroke, anticoagulation is recommended to be continued long-term after cardioversion according to the long-term anticoagulation recommendations, irrespective of the characterization of AF as a first-diagnosed episode [23]. However, although clinical outcomes of new-onset AF are less favorable than paroxysmal AF [36], prescription rates are the lowest in patients with first-diagnosed AF [37]. To date, no specific data regarding long-term anticoagulation in patients with new-onset AF and COVID-19 have been published. Thus, in our case series, the choice of long-term anticoagulation was always based on the CHA2DS2-VASc score and bleeding risk, as per the latest guidelines, and AF was never considered a mere infection-related epiphenomenon. DOACs were the preferred strategy in all cases, due to their favorable pharmacological profile, and physicians also based their clinical choice on the available data in this setting (although scarce), considering DOACs as the safest approach in managing non-valvular AF during the COVID-19 pandemic [38,39]. No significantly ischemic or hemorrhagic events were detected in the study cohort during follow-up.

- Regarding the antiarrhythmic strategy, a rate versus a rhythm control strategy was chosen depending on the managing physician’s choice and patient preferences as well. When considering patients who were still in AF during the post-discharge evaluation, the vast majority of patients were referred to a rhythm control strategy due to the well-known lower risk of adverse cardiovascular outcomes among patients with early AF treated with a rhythm control strategy, especially in cases of associated cardiovascular conditions [40]. The only case that was treated with a rate control strategy was an elderly patient who refused to be scheduled for an electrical cardioversion. Criteria for CA were based on a shared decision-making process, always summarizing baseline clinical characteristics and patients’ wishes. Age, comorbidities as well as AF burden (whenever possible in patients who were implanted with a loop recorder), and patients’ symptoms were always evaluated prior to CA. CA was offered whenever the managing physician thought CA would be the best treatment option, even in light of recent trials (EAST-AF-4 net, EARLY-AF, and STOP-AF) that have demonstrated the superiority of early rhythm control over rate control [40,41,42]. Of course, patients’ wishes were always taken into account, and only patients who had chosen CA as the best treatment option according to the benefit/risk ratio were treated with CA. Most patients who were treated with direct cardioversion were offered CA too, but they preferred to try to achieve rhythm control with a non-invasive strategy. Regarding the four cases who were referred for CA, physician choice was based on the presence of an underlying cardiac disease and/or on at least one cardiovascular risk factor, in order to improve clinical outcomes. Moreover, 3 out of 4 patients experienced at least one post-discharge AF recurrence (preferably detected with an ILR strategy, allowing a better recognition of arrhythmic episodes [43]), while one case was a persistent AF patient who experienced an early recurrence after an in-hospital electrical cardioversion. If it is indeed true that our small sample size does not allow us to make proper comparisons, no AF recurrences were detected in the post-CA group, while two recurrences were detected in the post-cardioversion group, corroborating recent evidence pointing towards the superiority of CA when compared to a drug-related rhythm control strategy in achieving freedom from AF, even in an early-AF setting [40,41,42,44]. Of note, as per current good clinical practice, all CA procedures were performed out from the infective state, at least 3 months after discharge.

### 4.3. Limitations

This case series presents several limitations. First, this was a purely retrospective analysis, significantly hampered by the small sample size. Second, several patients who might have developed AF during in-hospital admission might have not been referred to our arrhythmology clinic for a specific follow-up and therefore their clinical management strategy may have differed significantly when compared to our approach. Third, our small cohort did not include ICU patients, who are known to experience infection-related arrhythmia more frequently than non-severe patients due to several risk factors. Fourth, we did not include patients with new-onset AF during COVID-19 at home, which is very difficult to diagnose and examine. Fifth, despite including only patients that were not already been diagnosed with AF, we acknowledge that there may be a bias related to the inclusion of patients that may have already suffered from previous asymptomatic and unknown AF episodes at the time of the first evaluation. Sixth, we are not able to provide complete details of the vaccination status of all patients. Nevertheless, due to the wide enrollment timeframe, we expected that the vaccination status was very variegated, ranging from non-vaccinated patients hospitalized during the first phase, to fully vaccinated patients that were included later. Moreover, even if we acknowledge that the latest reported data are pointing towards a possible correlation between the COVID-19 vaccine and AF [45], these data come from an extremely large registry and thus such correlations would be impossible on our limited sample size. On the other hand, it should be underlined that we included only patients with new-onset AF detected during COVID-19 hospitalization, so that a link between new-onset AF and vaccination may be reasonably excluded.

## 5. Conclusions

Our data suggest that COVID-19 may trigger or reveal AF in vulnerable patients, who may frequently experience AF recurrences during follow-up. Therefore, AF should not be considered as a simple bystander of the in-hospital COVID-19 course. Management of new-onset AF post-COVID-19 patients referred to our specialistic electrophysiology clinic resembles our usual clinical practice, both in terms of long-term oral anticoagulation indications and in terms of rhythm control strategy.

## Figures and Tables

**Table 1:** Characteristics of the study cohort.

	Cohort (*n* = 23)
Age (years), mean ± s.d.	71.5 ± 8.1
Male, *n* (%)	20 (87.0)
Diabetes, *n* (%)	2 (8.7)
Hypertension, *n* (%)	14 (60.9)
Underlying cardiac disease, *n* (%) Ischemic cardiomyopathy, *n* (%) HFrEF, *n* (%)	2 (8.7) 2 (8.7) 1 (4.3)
Vascular disease, *n* (%)	3 (13.0)
LA volume index (ml/m^2^), median (IQR)	22 (18–24)
Moderate to severe MR, *n* (%)	1 (4.3)
History of stroke/TIA, *n* (%)	1 (4.3)
CKD, *n* (%)	2 (8.7)
CHA_2_DS_2_-VASc, median (IQR)	2 (1–3)
CHA_2_DS_2_-VASc < 2 (female) or < 1 (male), number of patients (%)	3 (13)
HAS-BLED, median (IQR)	2 (1–2)
Previous history of major bleeding, *n* (%)	1 (4.3)
Need for ICU hospitalization, *n* (%)	0 (0)
Need for CPAP during COVID-19 admission, *n* (%)	4 (21.7)
Anticoagulation at discharge, *n* (%) DOACs, *n* (%) LMWH, *n* (%)	21 (91.3) 19 (82.6) 2 (8.7)
Antiarrhythmic drugs at discharge, *n* (%) Amiodarone, *n* (%) Flecainide, *n* (%)	11 (47.8) 8 (34.8) 3 (13.0)
Cardioversion during admission, *n* (%)	16 (69.6)
VAs during COVID-19 admission, *n* (%)	1 (4.3)
Time from discharge to first ambulatory evaluation (days), median (IQR)	53 (41.5–127)
Follow-up time (days), median (IQR)	175 (83–336)
Patients monitored with an ILR, *n* (%) ILR implanted during admission, *n* (%) ILR implanted during post-discharge follow-up, *n* (%)	15 (65.2) 10 (43.5) 5 (21.7)

Abbreviations: CKD = chronic kidney disease; DOACs = direct anticoagulants; HFrEF = heart failure with reduced ejection fraction; ICU = intensive care unit; ILR = implantable loop recorder; IQR = interquartile range; LA = left atrial; LMWH = low-molecular-weight heparin; s.d. = standard deviation; MR = mitral regurgitation; TIA = transient ischemic attack.

## Data Availability

The data that support the findings of this study are available from the corresponding author, upon reasonable request.
